# Serum microRNA 143 and 223 Gene Expression Profiles as Potential Biomarkers in Individuals with Hepatitis and COVID-19

**DOI:** 10.3390/v16111734

**Published:** 2024-11-04

**Authors:** Lucas Lima da Silva, Luciane Almeida Amado Leon, Otacílio da Cruz Moreira, Wagner Luis da Costa Nunes Pimentel Coelho, Vanessa Duarte da Costa, Claudia Alexandra Pontes Ivantes, Priscila Pollo-Flores, Lia Laura Lewis-Ximenez, Vanessa Salete de Paula, Livia Melo Villar

**Affiliations:** 1 National Reference Laboratory for Viral Hepatitis, Institute Oswaldo Cruz, Fiocruz, Rio de Janeiro 21040-360, RJ, Brazil; v.duarte391@gmail.com (V.D.d.C.); llewis@ioc.fiocruz.br (L.L.L.-X.); 2 Technological Development Laboratory, Institute Oswaldo Cruz, Fiocruz, Rio de Janeiro 21040-360, RJ, Brazil; luciane.amado@gmail.com (L.A.A.L.); wagnerj97@hotmail.com (W.L.d.C.N.P.C.); 3 Molecular Virology and Parasitology Laboratory, Institute Oswaldo Cruz, Fiocruz, Rio de Janeiro 21040-360, RJ, Brazil; otacilio@ioc.fiocruz.br (O.d.C.M.); vdepaula@ioc.fiocruz.br (V.S.d.P.); 4 Gastroenterology, Hepatology and Liver Transplantation Service, Nossa Senhora das Graças, Curitiba 80810-040, PR, Brazil; claudiaivantes@gmail.com; 5 Department of Clinical Medicine, Fluminense Federal University, Niterói 24220-000, RJ, Brazil; priscilapollo96@gmail.com

**Keywords:** hepatitis, COVID-19, microRNA, biomarker

## Abstract

MicroRNAs (miRNAs) can act as biomarkers and descriptors of the association between infections and other diseases, such as hepatitis and COVID-19. This study aims to investigate the role of miRNA serum expression according to laboratory data concerning hepatitis and COVID-19. Seventy individuals recruited in Southern and Southeastern Brazil donated serum samples and were divided into four groups: (i) 20 negative subjects, (ii) 20 presenting hepatitis, (iii) 19 with COVID-19 and (iv) 11 with hepatitis and COVID-19. Three miRNAs (miR-122, miR-143 and miR-223) were evaluated using real-time PCR. Hematological and biochemical markers were also analyzed. MiR-143 and miR-223 were downregulated among the hepatitis/COVID-19 group (*p* < 0.05). A positive correlation was observed between miR-223 and lymphocytes. There was a negative correlation between alanine transaminase (ALT) and aspartate transaminase (AST) for miR-143 and miR-223 and gamma-glutamyl transferase (GGT), alkaline phosphatase (AP) and neutrophil/lymphocyte ratio (NLR) only for miR-223 (*p* < 0.05). For hepatic fibrosis (FIB-4), miR-122 and miR-143 had a greater association and miR-223 was more associated with a history of vaccination against COVID-19. MicroRNAs 143 and 223 could be useful as biomarkers for hepatitis coinfection with COVID-19.

## 1. Introduction

The disease caused by SARS-CoV-2 (COVID-19) is a systemic condition that affects various organs and tissues in the human body, including the liver [1]. Liver damage has been observed in COVID-19 cases, being present in half of patients hospitalized with COVID-19 [2] The proportion of developing liver damage in patients with severe COVID-19 was significantly higher than in mild cases and previous liver changes present in patients infected with SARS-CoV-2 have been considered a poor prognostic factor [3].

The definition of hepatitis is a condition that damages the liver, and can have different causes, such as different viral agents, autoimmune, drug-related or other conditions that can be related to inflammation, chronification and scarring lesions in the liver [4]. Among viral hepatitis, B and C play a major role in liver complications such as cirrhosis and hepatocellular carcinoma (HCC) [5], with more than 290 million chronic hepatitis B carriers globally and more than 50 million for chronic hepatitis C [6,7]. In addition to these viral factors, other pathologies are important contributors to liver damage associated with cirrhosis and HCC, including alcoholic steatohepatitis, where global data obtained in 2020 evidenced that 25% of deaths from liver cirrhosis were associated with high alcohol consumption [8] metabolic-associated steatotic liver disease (MASLD), with a 34% association in mortality and increased risk of HCC [9,10] and autoimmune hepatitis, with a high rate of cirrhosis among carriers, reaching more than 20% [11].

The challenge of understanding the processes involved in liver damage associated with COVID-19 has led to old questions and research into biomarkers that can help identify, characterize and prevent these diseases, which precede serious inflammatory conditions such as liver fibrosis, being a possible strategy for diagnosing the initial stage, carrying out treatment and preventing the development of more serious conditions such as HCC [5].

MicroRNAs (miRNAs) are non-coding small RNAs that can be investigated using fluids already used in other biochemical and serological tests for hepatitis, such as serum. They are single-stranded RNA molecules of approximately 19–25 nucleotides, which serve as potent post-transcriptional regulators of gene expression playing a regulatory role by binding to the 3′ untranslated region of target messenger RNAs (mRNAs) [12]. Recent studies revealed their importance by describing alterations in the gene expression of miRNAs in different diseases, in which some miRNAs can act as biomarkers [5,13].

To pathological conditions, different miRNAs are studied in relation to specific physiological disorders, such as liver diseases. In this case, miRNAs can be expressed with high or low regulation when assessed for pre-disposition to liver fibrosis and can act as an indicator of the initial stages of tissue scarring in the liver, being a promising biomarker [14,15]. This presents advantages when compared to other methods, as it has greater sensitivity and high diagnostic accuracy, and can be used for early detection as well as monitoring the progression of the disease and response to treatment. Despite this, they are highly variable to several physiological factors, biologically complex and require high standardization and analysis costs [16].

Hepatocyte-derived miR-122 has a high association with liver damage, is liver-specific and correlates strongly with liver enzyme levels and necro inflammatory activity. It is also an important biomarker and previously known cofactor for hepatitis C, with high expression during infection, and is currently being used as a therapeutic target; despite this, during the progression of fibrosis and HCC, the expression of this miRNA is reduced, specifically for hepatitis B [5,15]. MiR-122 was evaluated for binding to SARS-CoV-2 with high affinity, thus suggesting a redirection of action of anti-HCV RNA-based drugs that used this miRNA against hepatitis C virus (HCV) towards SARS-CoV-2 [13].

MiR-143 is encoded by chromosome 5q32 and expressed in various tissues, but with low expression when related to different tumors [17]. This reduced expression has also been related to cases of hepatitis B associated with fibrosis progression [18]. Combinatorial multi-omics profiling/genotyping and miRNA and RNA expression analyses identified that miR-143 influences the neutrophil count associated with the inflammatory condition of COVID-19 [19]. MiR-223 is encoded by the q12 locus on the X chromosome in humans and is involved in the regulation of various transcription factors, mainly by acting as an inflammatory mediator, with implication in the regulation of monocyte–macrophage differentiation, neutrophil recruitment and pro-inflammatory responses, this being its direct relationship with liver diseases [20]. Studies have suggested that serum levels of this miRNA decrease in relation to the progression of liver fibrosis in patients with hepatitis B [20,21]. The role of this miRNA in regulating inflammation may suggest that it plays a role in the immunopathogenesis of COVID-19 [22].

The aim of this study was to characterize the serum expression of miR-122, miR-143 and miR-223 in relation to the presence of SARS-CoV-2 infection and the association with liver disease in order to characterize and associate the expression of these biomarkers with individuals with hepatitis and COVID-19.

## 2. Materials and Methods

### 2.1. Patient Selection and Collection of Biological Material

This study was approved by the ethics committee of Oswaldo Cruz Foundation under approval number 11177119.8.0000.5248. Seventy individuals were selected from a cohort of two medical care units located in Southern and Southeastern Brazil (Rio de Janeiro and Paraná states) from which four homogeneous groups were stratified in terms of age, gender and comorbidities. For homogeneity between the groups, comorbidities that could alter the expression of miRNAs in relation to liver disease and COVID-19 were removed; these included cancer, diabetes and hypertension. The groups were divided into 20 control individuals without hepatitis and/or COVID-19 (group 1), 20 individuals with hepatitis (group 2), 19 individuals with COVID-19 (group 3) and 11 individuals with hepatitis and COVID-19 (group 4). Serum and naso-oropharyngeal samples were collected from these individuals intravenously and stored in cryotubes at −70 °C. All patients completed a consent form, and a standardized questionnaire was used to collect sociodemographic data.

### 2.2. Detection and Diagnosis of Hepatitis

The definition of liver conditions was based on the clinical assessment of patients through a review of medical records carried out by the healthcare team. The patient care units employed the diagnosis of viral hepatitis using criteria established by the European Association for the Study of the Liver guidelines for hepatitis B, C, D and E [23,24,25,26] and guidelines for the diagnosis of hepatitis A established by the Centers for Disease Control and Prevention [27]. In this study, we included only individuals with reactivity to hepatitis B surface antigen or antibodies against hepatitis C, who were considered HBV and HCV individuals. For the diagnosis of nonviral hepatitis, the criteria were based on the Revised and Adapted Score for the Diagnosis of Autoimmune Hepatitis [28] or the Lindor criteria of cirrhosis [29]. As for steatosis, diagnostic methods such as ultrasonography and tomography were evaluated to identify the degree of fatty deposits in the liver [30]. To establish advanced fibrosis, the non-invasive Fibrosis-4 (FIB-4) index method was used, based on hematological and biochemical markers and the age of the individuals, using the METAVIR score, where F0–F1 defines absence of fibrosis or mild fibrosis, F2 moderate fibrosis and F3–F4 advanced fibrosis, characteristic of cirrhosis [31,32].

### 2.3. Detection and Diagnosis of COVID-19

Viral RNA from swab samples was extracted with the QIAamp Viral RNA Mini Kit (QIAGEN, Hilden, Germany). After extracting the viral RNA, the RT-qPCR methodology was employed using the commercial reagent “AgPath-ID^TM^ One-Step RT-PCR” (Thermo Fisher Scientific, Waltham, MA, USA) with a final volume of 25 µL containing 5 µL of extracted RNA and 20 μL of the reaction mixture. Oligonucleotides associated with probes (assays) targeting regions of the SARS-CoV-2 genome were used in the assay as described previously [33,34]. The amplification targets corresponded to the viral nucleocapsid (N1 and N2), from the protocol provided by the CDC, and the envelope (E) from the protocol published by Corman et al. [34]. The reaction was conducted using the Rotor-Gene Plex 5 (QIAGEN, Hilden, Germany), with probes detected in the FAM channel. According to these protocols, Cycle Thresholds (CT) below 35 indicated a virus was detected (positive) and above 40 indicated one was not detected (negative). Between 35 and 40 requires confirmation. To detect positivity, it was established that two regions should be identified in the qPCR.

### 2.4. MiRNA Isolation, Complementary DNA (cDNA) Synthesis and Quantification in Real-Time PCR

RNA was isolated from 200 µL of serum samples using a commercial extraction kit named miRNeasy Serum/Plasma Kit (QIAGEN). Five microliters of cell-miR-39 (Life Technologies, Carlsbad, CA, USA—ASSAY ID: 000200), sequence 3pUCACCGGGUGUAAAUCAGCUUG, at a concentration of 1 fmol/µL was added to RNA extraction for normalization and control of subsequent tests using quantitative reverse transcription polymerase chain reaction (RT-qPCR). The final product of the isolation was distributed in 5 µL for cDNA synthesis with target-specific stem–loop primers, based on standardized protocols using the TaqMan MicroRNA Reverse Transcription Kit (Applied Biosystems, Beverly, MA, USA). The final reaction volume for cDNA synthesis was 15 µL for each individual target. After synthesizing the cDNA, a qPCR test was carried out using TaqMan^TM^ Universal Master Mix II with UNG (Thermo Fisher Scientific, Waltham, MA, USA) and specific TaqMan miR-specific assays (Applied Biosystems^®^) comprising hsa-miR-122-5p (Assay ID: 002245), hsa-miR-143-3p (Assay ID: 002249) and hsa-miR-223-3p (Assay ID: 002295). The distribution on the real-time PCR plate (0.1 mL) was 9.33 µL of the mix containing Master Mix, the assay for a specific target and water and 0.67 µL of cDNA. The conditions for amplifying the genetic material using the QuantStudio 3 apparatus (Thermo Fisher Scientific) were as follows: 50 °C for 2 min for Uracil N-Glycosylase (UNG) activation, 95 °C for 10 min for TaqMan activation, 95 °C for 15 s for denaturation and 60 °C for 1 min for hybridization/extension, the last two in 40-fold cycles. All samples were amplified in duplicate and a threshold of 0.02 was set for all targets. The average values of Ct, ΔCt, ΔΔCT and relative quantification (RQ) were calculated [35]. The expression of the examined miRNAs’ fold change was calculated by relative quantification (comparative Ct method, with assessment of miRNA expression, comparing it to a reference), using the formula RQ = 2^−ΔΔCT^.

### 2.5. Biochemical and Hematological Tests

For the biochemical tests for alanine transaminase (ALT), aspartate transaminase (AST), gamma-glutamyl transferase (GGT), alkaline phosphatase, total bilirubin and fractions, glucose and C-reactive protein (CRP), the dry chemistry methodology was used using the Clinical Chemistry Analyzer AU680 (Beckman Coulter, CA, USA). The hematological tests were carried out using flow cytometry and based on the electrical impedance methodologies recommended for the Coulter LH 750 Hematology Analyzer (Beckman Coulter). To assess severity, the following ratios between biochemical markers were used: neutrophil/lymphocyte ratio (NLR), monocyte/lymphocyte ratio (MLR) and platelet/lymphocyte ratio (PLR).

### 2.6. Statistical Analysis

GraphPad Prism 9 and RStudio were used to analyze the data and construct the figures and graphs. For expression analysis and graph construction, quantification cycle (Cq) values were used; for statistical analysis and comparison between groups, ΔCq values were used, employing the Mann–Whitney test, according to non-parametric description between groups. Significance was set at *p* < 0.05. A cut-off of 1.5 was set for the association between the hematological and biochemical variables and the fold change values, establishing high and low expression of the targets analyzed. The chi-square test and Fisher’s correction were used to assess the significance between the expression of miRNAs and the variables analyzed. The receiver operating characteristic (ROC) curve was established to assess sensitivity and specificity with the variables that presented significance in the chi-square test. Pearson’s test was used to correlate expression with hematological and biochemical markers.

## 3. Results

### 3.1. Study Population

Demographic and laboratory data from the study population is presented in Table 1. The mean age was 49 years (±14.67), and 37 (53%) were women. Of the 31 individuals with liver disease, 19% (6/31) had chronic hepatitis B, 26% (8/31) chronic hepatitis C, 49% (15/31) hepatic steatosis, 3% (1/31) cirrhosis due to alcohol consumption and 3% (1/31) cirrhosis due to autoimmune liver disease. The hepatitis/COVID-19 group had a higher mean of laboratory markers related to inflammation when compared to other groups. There was a mean of 529 mm^3^ (±915.5) for band cells, 36.48 mg/dL (±SD 93.97) for C-reactive protein and 118 U/L (±SD 115) For alkaline phosphatase. Biochemical markers of liver function were higher among the individuals with hepatitis, with a mean of 86 U/L (±SD 125) for alanine aminotransferase and 151 UI/L (±SD 169) for gamma-glutamyl transferase in the hepatitis-only group.

### 3.2. Serum miRNA Expression in Relation to Groups

**Figure 1:**
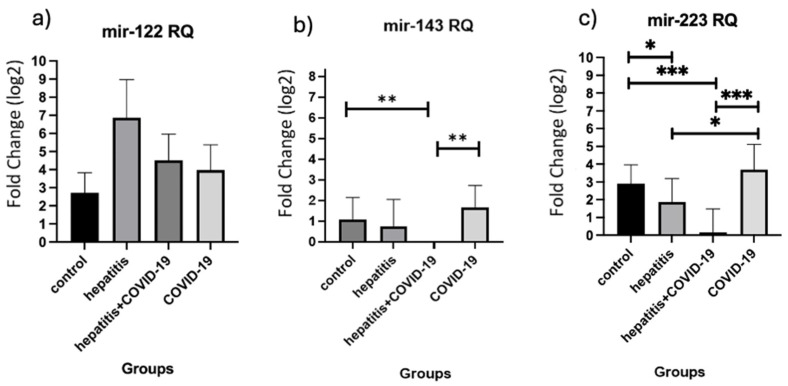
Expression level for each target analyzed. (**a**) Fold change (RQ levels in log2) and statistical analysis using ΔCt for target miR-122; (**b**) fold change (RQ levels in log2) and statistical analysis using ΔCt for target miR-143; (**c**) fold change (RQ levels in log2) and statistical analysis for target miR-223. The asterisks (*) represent statistical significance where *: *p* = 0.05; **: *p* = 0.01 and ***: *p* = 0.001.

MiRNA serum expression was analyzed and the relationship between the groups is presented in Figure 1. A higher expression of miR-122 in the hepatitis-only and hepatitis + COVID-19 groups when compared to other groups can be observed. Statistical significance was found for the lower expression of miR-143 in the hepatitis + COVID-19 group when compared to control (RQ mean = 0.55 vs. 2.10, *p* = 0.0094) and COVID-19-only (RQ mean = 0.55 vs. 3.19, *p* = 0.0037) groups. MiR-223 showed a lower expression in groups composed of liver disease patients, with or without COVID-19. Statistical significance was observed for the hepatitis-only group when compared to control (RQ mean = 3.67 vs. 7.52, *p* = 0.0406) and COVID-19-only (RQ mean = 3.67 vs. 12.94, *p* = 0.0268) groups. The same was observed in the hepatitis + COVID-19 group when compared to control (RQ mean = 1.1 vs. 7.52, *p* = 0.0009) and COVID-19-only (RQ mean = 1.1 vs. 12.94, *p* = 0.0006) groups.

### 3.3. Correlation Between miRNA Expression and Hematological and Biochemical Markers

**Figure 2:**
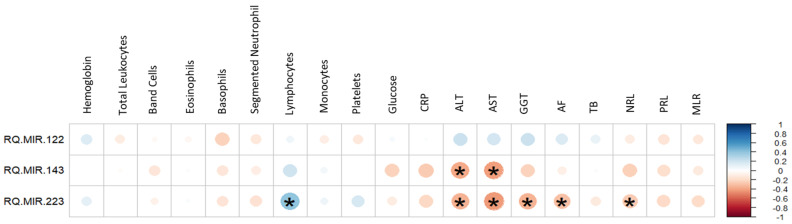
Pearson’s correlation for miRNA expression analysis (fold change) and the hematological and biochemical markers analyzed in the study. The heat map indicates a negative correlation with red circles; the higher the level of fold change (RQ), the lower the values of the hematological markers. The positive correlation is shown in blue circles. CRP: C-reactive protein; ALT: alanine aminotransferase; AST: aspartate aminotransferase; GGT: gamma-glutamyl transferase; AF: alkaline phosphatase; BT: total bilirubin; NRL: neutrophil lymphocyte ratio; PLR: platelet lymphocyte ratio; MLR: monocyte platelet ratio.

The correlation between miRNA expression and hematological and biochemical markers is presented in Figure 2. A positive relationship between lymphocytes and the expression of miR-223 (*p* = 0.001) was observed. On the other hand, ALT and AST were indicated as having a negative correlation with miR-143 (*p* = 0.002 and *p* = 0.0005, respectively) and miR-223 targets (*p* = 0.004 and *p* = 0.0003, respectively). There was also a negative correlation for miR-223 with GGT, alkaline phosphatase (AF) and neutrophil lymphocyte ratio (NRL) (*p* = 0.005, *p* = 0.018 and *p* = 0.03, respectively).

### 3.4. Evaluation of Sensitivity and Specificity of miRNAs and Significant Variables

**Figure 3:**
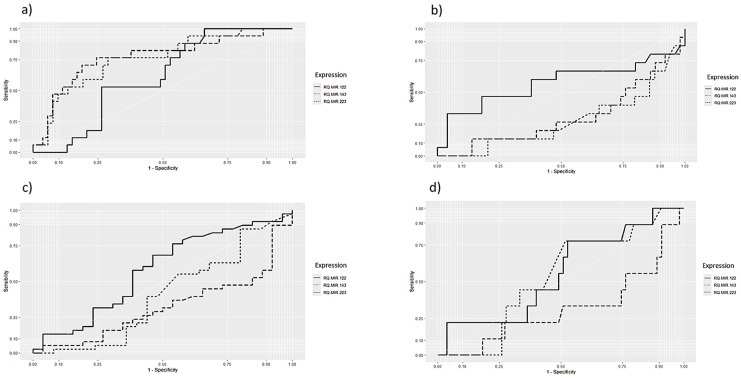
ROC curve based on the variables that showed statistical significance in the chi-square test for association with the miRNAs analyzed: (**a**) COVID-19 vaccine; (**b**) AST; (**c**) GGT; (**d**) Fibrosis-4 (FIB-4).

Figure 3 shows the ROC curve evaluating sensitivity and specificity between the expression of miRNAs and the significant variables in the chi-squared test. For COVID-19 vaccination history (a), it was observed that the expression of the miR-223 target suggested higher sensitivity and specificity values with AUC: 0.770 (sensitivity: 0.764—specificity: 0.754). For AST (b—AUC: 0.574 sensitivity: 0.333—specificity: 0.960) and GGT (c—AUC: 0.581 sensitivity: 0.763—specificity: 0.461), miR-122 showed higher sensitivity and specificity when compared to the other miRNA targets. For liver fibrosis (d), miR-122 (AUC: 0.556 sensitivity 0.777—specificity: 0.472) and miR-143 (AUC: 0.530 sensitivity 0.777—specificity: 0.481) presented similar values for the sensitivity/specificity ratio.

## 4. Discussion

In this study, the expression of miRNAs 122, 143 and 223 was observed in relation to SARS-CoV-2 infection in patients with a history of liver diseases. Our results demonstrate a downregulation of these three targets in the hepatitis/COVID-19 group when compared to the other groups, corroborating our hypothesis that some alteration would be found in these individuals when compared to the others with only COVID-19 or only hepatitis. This specific variation in this group can be presented as a biomarker that characterizes the profile of the association between COVID-19 and hepatitis caused by other agents, viral or non-viral. When we analyze each of the miRNA targets in a stratified format, we find that miR-122 has a higher expression among the groups with hepatitis. This miRNA is known in the literature for its high production in liver tissue (around 70%) and its important role in HCV replication, with high expression in these individuals [36]. The same is present in individuals with hepatitis B, where it is upregulated at around 1.5-fold [37]. In cases of non-viral hepatitis and individuals with alcoholic fatty liver disease, it was found that downregulation of this miRNA protects hepatocytes from lipid accumulation [38], which corroborates our findings that upregulation is associated with a history of liver disease.

For miR-143, there was a low expression in the hepatitis/COVID-19 group when compared to the control and COVID-19-only groups. This is a miRNA expressed in different tissues and a tumor suppressor encoded by chromosome 5q32 [18]. It has an important role in the aggravation of viral hepatitis B and C and is downregulated in chronic hepatitis B with fibrosis progression [21]. Previous studies have found similarly low values of this miRNA expression in the serum of patients with hepatitis C associated with HCC when compared to control [17]. The same was observed in the serum of cirrhotic nonviral hepatitis patients, associated with HCC, when compared to control [17]. In relation to COVID-19, a study that evaluated the profile of patients with SARS-CoV-2 infection in relation to abnormalities in blood coagulation observed an upregulation of this miRNA expressed in the serum of infected patients when compared to the control group [39].

In this study, miR-223 downregulation was observed for the hepatitis/COVID-19 group when compared to the other groups. It originates from the q12 locus on the X chromosome and is involved in different pathological conditions, with a high immunological and inflammatory relation factors, which connects it with liver diseases [20]. Serum expression of this miRNA is downregulated during hepatitis B virus (HBV) infection, including in conditions associated with fibrosis progression and HCC [21]. However, the opposite happens in individuals with HCV associated with progression of fibrosis and cirrhosis, where there is an upregulation of this miRNA in serum [40]. In cases associated with HCC, downregulation occurs [41]. A study evaluating patients with COVID-19 in relation to pulmonary diseases demonstrated a low miR-223 expression in patients with pulmonary fibrosis-like lesions, with low expression in this group [42]. In this study, we found an upregulated expression in the COVID-19-only group, which may represent a notable role in the clinical diagnosis of these individuals. These results highlight the downregulation of miR-143 and miR-223 among the individuals with hepatitis/COVID-19 in this study, which may represent the presence of SAR-CoV-2 infection in the downregulation of these targets. The characterization of these expressions of miRNAs plays an important role in predicting clinical worsening associated with COVID-19 and hepatitis, as well as representing a promising therapeutic alternative based on the application of these genetic targets to different diseases [43].

In the correlation between the miRNAs analyzed and the biochemical and hematological markers, a positive correlation was observed between miR-223 and lymphocytes. This is a miRNA directly related to inflammatory conditions, playing a role in the regulation of biological processes and immune modulation. A study showed that the expression of miR-223 was positively regulated in CD4 T lymphocytes during pathological conditions [44]. Another study showed that this miRNA is highly expressed in lymphocytes and participates in differentiation and proliferation in autoimmune diseases [45]. In the lung, miR-223 acts as an important factor in acute lung injury by regulating the NOD-like receptor protein 3 (NRLP3) inflammasome [46].

A negative correlation was observed between the ALT and AST markers for the miR-143 and miR-223 targets, the latter also having negative correlation to GGT, AF and NLR. The opposite has been observed in the literature. A study evaluated the expression of miR-143 in individuals with autoimmune hepatitis as a cause of fibrosis and found a positive correlation with the liver biochemical markers ALT and AST [47]. Another study showed a positive correlation between miR-223 and ALT and AST in those predisposed to severe liver disease [48]. The impact of COVID-19 on the study population may be affecting these correlations. The profile of the individuals studied is mostly mild/moderate liver disease, with normal levels of biochemical markers and without the presence of severe COVID-19. Despite the mild/moderate profile of the individuals in our study, the association between these miRNAs and hematological and biochemical markers may contribute to understanding the clinical signs and symptoms present in SARS-CoV-2 infection in patients with liver disease and justify advanced hospitalization and care. There are no studies that directly associate miR-223 with GGT or AF, which demonstrates the authenticity of this work. For NLR, this is the miRNA directly involved in the immune and inflammatory response, as well as the production of defense cells such as neutrophils [22].

Based on this immunological principle, the ROC curve showed higher specificity and sensitivity for miR-223 associated with individuals’ COVID-19 vaccination history [49]. It is known that the vaccine interferes with the expression of miRNAs and this specifically, due to its immunological role, is consistent with an expected vaccine immune response after the application of the immunizer and the production of immunological memory and can act as a biomarker indicating an adequate vaccine response [50].

As for the hepatic biochemical variables TGO and GGT, miR-122 showed better sensitivity and specificity in the ROC curve. As this miRNA is highly produced in the liver and is directly associated with liver problems, it is a good biomarker for predisposition to and description of liver disease by associating it with liver biochemical markers [5,37,51].

For liver fibrosis, miR-143 and mir-122 showed good sensitivity and specificity values in the ROC curve. From this study, these are the two miRNAs most associated with the liver. The highlight for miR-143 is that this miRNA has already been described in several studies due to its relation to liver tissue cicatrization, characteristic of fibrosis, making it an important biomarker in the characterization and investigation of diseases that can cause this condition [17,18,47]. The levels of sensitivity and specificity found in this study when associated with the expression of miRNAs are evidence of a direct correlation between these targets and the clinical condition of individuals, and may act as predisposing factors for more serious conditions such as liver fibrosis, which can be investigated before high degrees of cicatrization and loss of liver tissue are identified. To conclude this hypothesis, there is a need to follow up with these individuals and measure the expression of these miRNAs again after identifying damage to liver tissue. In any case, this does not exclude the association found in this study and the role of miRNA biomarkers in characterizing the association between hepatitis and COVID-19. This study has limitations in terms of the uneven distribution of individuals between the groups, which reduced the number of people in each group. Despite this, this study presents a total number of individuals consistent with the line of exploratory research [52].

## 5. Conclusions

In conclusion, all the miRNAs analyzed had different expression among the hepatitis/COVID-19 group when compared to the other groups in the study. MiR-143 and miR-223 were downregulated in this group, with statistical significance. This demonstrates the impact of the association between COVID-19 and a history of liver disease on the expression of these miRNAs, as well as the role of these biomarkers in characterizing this association. The correlations between these miRNA targets and the hematological and biochemical markers define the characterization of the clinical laboratory status of the individuals included in the study and demonstrate the authenticity of this work in relation to the literature in an attempt to understand the signs and symptoms associated. It was observed in this study that miRNAs can act as biomarkers with levels of sensitivity and specificity for variables that are associated with their synthesis and action. MiR-122, highly produced in the liver, had high levels of sensitivity and specificity for the biochemical markers TSO, AF and fibrosis; MiR-143, with regulation of its expression associated with tissue cicatrization, had high sensitivity and specificity for liver fibrosis; and miR-223, with its expression and synthesis associated with the immune response, had high sensitivity and specificity for the history of vaccination by COVID-19.

## Figures and Tables

**Table 1:** Demographic and laboratory characteristics of the study population.

Characteristics	Control (n:20)	Hepatitis (n:20)	COVID-19 (n:19)	Hepatitis + COVID-19 (n:11)	Total (n:70)
**Mean Age (±SD)**	47 (±15.87)	51 (±13.49)	47 (±14.34)	50 (±16.32)	49 (±14.67)
**Gender (%)**					
Male	11 (55)	9 (45)	6 (32)	7 (64)	33 (47)
Female	9 (45)	11 (55)	13 (68)	4 (36)	37 (53)
**Mean laboratory markers (±SD)**					
Hemoglobin g/dL	13.67 (±1.74)	13.61 (±1.33)	13.82 (±1.39)	13.54 (±1.94)	13.67 (±1.53)
Leukocytes mm	6.437 (±1.403)	6.214 (±2.498)	12.136 (±2.207)	5.546 (±3.092)	7.739 (±11.590)
Band Cells mm^3^	143.9 (±63.73)	413.5 (±620.5)	133 (±66)	529 (±915.5)	270 (±484)
Eosinophils mm^3^	156.81 (±205.9)	144.4 (±204.4)	32 (± 51.8)	125 (±134.3)	115 (± 172)
Basophils mm^3^	6.35 (±19.8)	4.42 (±13.09)	4.9 (±21.47)	0 (±0)	4.7 (±17.4)
Segmented neutrophils mm^3^	3.572 (± 1.246)	3.865 (±2.215)	4.510 (±2.530)	3.708 (±1.894)	3.936 (±2.012)
Lymphocytes mm^3^	2.070 (± 815)	1.325 (±698)	1.765 (±890)	1.148 (±660)	1.657 (±845)
Monocytes mm^3^	408.5 (±136.1)	397.11 (±180.19)	543.94 (±305.33)	493.76 (±318.39)	425.63 (±232.62)
Platelets mm^3^	255.050 (±520.38)	211.895 (±891.20)	270.471 (±799.54)	308.375 (±267.535)	253.000 (±116.812)
Glucose mg/dL	100 (±36)	101 (±33)	121 (±64)	117 (±34)	108 (±44)
C-reactive protein mg/L	0.49 (±0.51)	9.5 (±21.7)	3.9 (±6.09)	36.48 (±93.97)	9.13 (±37.09)
Alanine aminotransferase U/L	35 (±15)	86 (±125)	28 (±17)	44 (±10)	48 (±72)
Aspartate aminotransferase U/L	20 (±7)	49 (±35)	27 (±25)	46 (±28)	35 (±27)
Gamma-glutamyl transferase U/L	43 (±29)	151 (±169)	49 (±50)	92 (±75)	82 (±109)
Alkaline phosphatase U/L	20 (±26)	106 (±61)	70 (±22)	118 (±115)	87 (±57)
Total bilirubin mg/dL	0.65 (±0.44)	1.01 (±0.84)	0.51 (±0.20)	1.17 (±0.71)	0.77 (±0.60)

SD: standard deviation.

## Data Availability

Data are contained within the article.
